# Diagnostic value of CT scans in pediatric patients with acute non-traumatic altered mental status: a systematic review and meta-analysis

**DOI:** 10.1007/s00431-024-05943-3

**Published:** 2025-01-15

**Authors:** Mohammed Alsabri, Mohammed Ayyad, Mayar M. Aziz, Mohamed Sayed Zaazouee, Alaa Ahmed Elshanbary, Muhammad Ashir Shafique, Lamar Sarieddine, Ibrahim Qattea, Muhammad Waseem, Luis L. Gamboa

**Affiliations:** 1 Pediatric Emergency Department, St. Christopher’s Hospital for Children, Drexel University College of Medicine, Philadelphia, PA USA; 2 Department of Internal Medicine, Rutgers New Jersey Medical School, Newark, NJ USA; 3 Faculty of Medicine - Menofia Universit, Menofia Governorate, Egypt; 4 Faculty of Medicine, Al-Azhar University, Assiut, Egypt; 5 Faculty of Medicine, Alexandria University, Alexandria, Egypt; 6 Jinnah Sindh Medical University, Karachi, Pakistan; 7 University of Balamand, Beirut, Lebanon; 8 Pediaitric department, Nassau University Medical Center, Nassau, NY USA; 9 Emergency Medicine, Lincoln Medical Center, Bronx, NY USA

**Keywords:** Computed tomography, Pediatric, Altered mental status, Meta-analysis, Diagnostic yield

## Abstract

**Background:**

Computed tomography (CT) scans are widely used for evaluating children with acute atraumatic altered mental status (AMS) despite concerns about radiation exposure and limited diagnostic yield. This study aims to assess the efficacy of CT scans in this population and provide evidence-based recommendations.

**Methods:**

A systematic review was conducted according to PRISMA guidelines. Comprehensive searches were performed in PubMed, Embase, Cochrane Library, Scopus, and Web of Science for studies involving pediatric patients with acute atraumatic AMS undergoing head CT scans. Two independent reviewers conducted the literature search, extracted data, and assessed study quality.

**Results:**

From 4,739 identified studies, 13 met the inclusion criteria. The overall positive diagnostic yield of head CT scans was 35.9% (95% CI: 6.1%–65.7%). Subgroup analyses revealed that the diagnostic yield varied by clinical setting, age group, and presenting symptoms.

*Conclusion*: Head CT scans are frequently performed in pediatric patients with AMS, but their diagnostic usefulness is limited. Evidence-based guidelines and risk stratification methods are necessary to improve imaging utilization and minimize radiation exposure risks.

**Table:** 

What is Known • *Computed tomography (CT) scans are commonly used to evaluate pediatric patients with acute atraumatic altered mental status (AMS).* • *There are concerns about radiation exposure from CT scans, especially in children due to their increased sensitivity and longer life expectancy.* • *Previous studies suggest a low diagnostic yield of CT scans in certain pediatric conditions, indicating potential overuse.*
What is New • *This systematic review and meta-analysis specifically assess the diagnostic value of CT scans in pediatric patients with acute atraumatic AMS.* • *Findings reveal a relatively low positive diagnostic yield, indicating that CT scans may be overutilized in this population.* • *Subgroup analyses highlight variability in outcomes based on clinical setting, patient age, and presenting symptoms.* • *The study underscores the need for evidence-based guidelines and risk stratification tools to optimize imaging decisions and reduce unnecessary radiation exposure in children.*

**Supplementary Information:**

The online version contains supplementary material available at 10.1007/s00431-024-05943-3.

## Introduction

Computed Tomography (CT) is a widely used neuroimaging procedure for evaluating pediatric patients with acute atraumatic altered mental status (AMS), a term encompassing clinical presentations such as confusion, disorientation, and decreased responsiveness. AMS in children can arise from various causes, including infections, metabolic disorders, seizures, and neuroinflammatory conditions, with CT imaging being particularly common in emergency department evaluations for seizures or epilepsy [1, 2].

While CT scans are diagnostically valuable, concerns persist about the potential malignancy risks associated with ionizing radiation exposure. Studies indicate that moderate doses of 50–60 mGy may increase the risk of brain neoplasms [3, 4]. The U.S. Food and Drug Administration (FDA) has stated that no radiation dose is entirely risk-free, emphasizing the need for judicious use of CT imaging [5, 6]. The frequency of CT use in pediatric AMS varies by healthcare setting, with general emergency departments using CT scans more frequently than specialized pediatric emergency departments [7]. However, a retrospective study found that only a small proportion of children undergoing CT scans for their first seizure had abnormal findings, with even fewer requiring clinical intervention [8, 9].

Clinical guidelines, such as those from the American Academy of Neurology, recommend limiting CT scans to specific conditions, including persistent post-ictal neurological deficits lasting more than 36 h or abnormal neurological status following a seizure [10]. Despite these recommendations, adherence to these guidelines is inconsistent across clinical settings. Studies evaluating children with epilepsy reveal that many CT scans are performed without meeting the criteria for urgent imaging, reflecting a gap between evidence-based recommendations and clinical practice [11, 12].

Previous research, including a meta-analysis by Mower et al., assessed the use of CT imaging in patients with acute atraumatic AMS but included a broad age range and lacked a pediatric-specific focus [13]. Given children’s increased susceptibility to the risks of ionizing radiation, there is a pressing need to evaluate the diagnostic value of CT scans exclusively in pediatric patients. This study aims to assess the diagnostic yield of CT imaging in pediatric AMS and provide evidence-based recommendations to optimize imaging practices and minimize unnecessary radiation exposure.

## Methods

This meta-analysis was conducted in accordance with the PRISMA guidelines and adhered to the Cochrane Handbook for Systematic Reviews and Meta-Analyses of Interventions [14, 15]. The study protocol was prospectively registered with PROSPERO under the registration number CRD42023482992.

## Information sources and search strategy

A systematic search of PubMed, Embase, Cochrane Library, Scopus, and Web of Science was performed to identify relevant studies published up to May 2024. Search terms included combinations of "pediatric," "children," "computed tomography," "altered mental status," "diagnosis," and "outcomes" (Supplementary Table S1). Additional manual searches of reference lists from included studies were performed to identify potentially eligible studies.

## Eligibility criteria

Studies were included if they:

1. Investigated pediatric patients (< 18 years) presenting with acute atraumatic AMS and undergoing head CT scans.
2. Reported data related to clinical outcomes, diagnostic methods, management strategies, or presenting symptoms.
3. Provided sufficient data for extraction and analysis.

Excluded studies were reviews, abstracts, letters to the editor, non-peer-reviewed articles, and those with insufficient data. Studies involving adult populations or mixed populations without separate pediatric data were also excluded.

## Selection process

Duplicates were removed using EndNote software. Two independent researchers screened titles and abstracts for relevance, followed by full-text reviews for eligibility. Disagreements were resolved by consensus or consultation with a third reviewer. The Rayyan platform facilitated the selection process.

## Data collection process and data items

Data were extracted independently by two reviewers using a standardized form. Extracted data included study characteristics (author, year, design), patient demographics (age, sex), clinical presentations (symptoms, diagnostic methods), treatment strategies, and clinical outcomes (mortality, complications, recurrence of AMS). Discrepancies were resolved through discussion or consultation with a third reviewer.

## Definition of positive diagnostic yield of head CT

Positive diagnostic yield of head CT was defined as scans revealing clinically significant findings influencing patient management, such as intracranial hemorrhage, mass lesions, or other acute abnormalities.

## Risk of bias assessment

The risk of bias was assessed using the ROBINS-I tool, which evaluates key domains such as confounding, participant selection, intervention classification, missing data, and outcome reporting. Each domain was rated as 'low risk,' 'moderate risk,' or 'serious risk.' Two assessors independently evaluated bias, resolving disagreements by consensus.

## Statistical analysis

Data were synthesized using a random-effects meta-analysis to pool event rates and account for variability between studies. The primary outcomes were reported as pooled event rates with 95% confidence intervals (CIs). Heterogeneity was assessed using the I^2^ statistic, with I^2^ values above 50% indicating substantial heterogeneity. The Chi-square test (Cochrane Q test) was used to test the significance of heterogeneity (*p* < 0.1 considered significant).

Subgroup analyses were performed to assess diagnostic yield differences by clinical setting (emergency department vs. inpatient), age groups (< 1 year, 1–12 years, 13–18 years), gender, and presenting symptoms (e.g., seizures, headaches, focal neurological deficits).

Publication bias was assessed through funnel plots, and sensitivity analyses were conducted to evaluate the robustness of pooled estimates by excluding studies at high risk of bias. Statistical analyses were conducted using Review Manager (RevMan, version 5.4) and R software (version 4.2.1

## Results

**Fig. 1:**
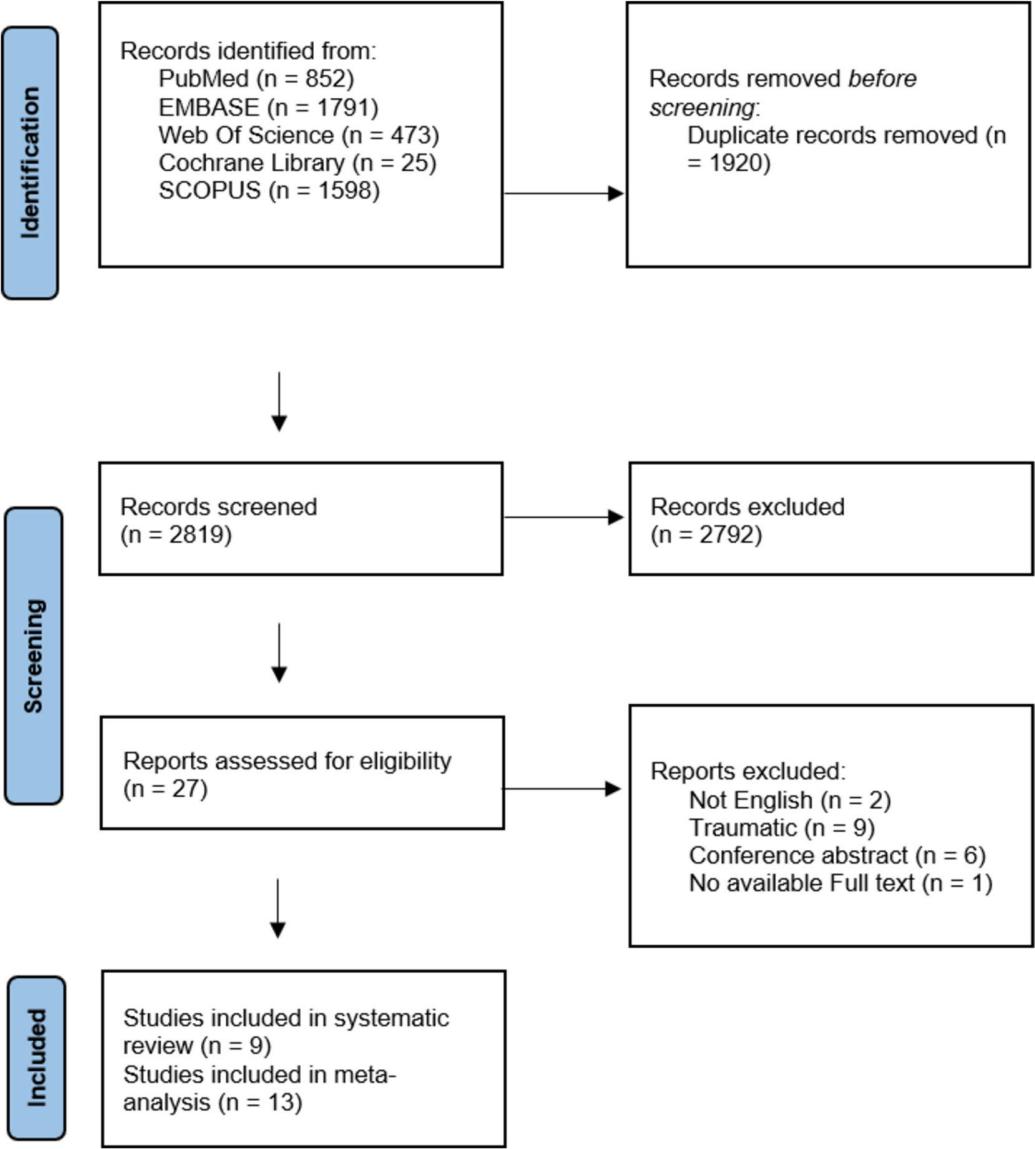
PRISMA diagram of the included studies

Our literature search process retrieved 4,739 records. After screening titles and abstracts, 2,819 articles were identified as potentially relevant for full-text screening. Of these, 13 observational studies were included in the systematic review, and 9 studies were incorporated into the meta-analysis. The PRISMA flow diagram summarizing the study selection process is shown in Fig. 1.

Thirteen observational studies, comprising a total of 2,629 pediatric patients with acute atraumatic AMS, were included in this meta-analysis. Key characteristics of the included studies, including study design, patient demographics, clinical outcomes, and treatment interventions, are summarized in Table 1 and Table 2.

**Table 1:** Summary of included studies

Study ID	Design	Country	Duration	Sample Size	Patients Age Group	Intervention	Indication of CT
Allen et al. 2007	Retrospective study	US	2000–2001 and 2003–2004	124	Pediatrics	Computed tomography of the head (CTH)	Seizure, head trauma, headache, and shunt malfunction
Cavallaro et al. 2021	Retrospective study	US	2006 to 2017	5.41 million	Pediatrics	CT at pediatric emergency department	Seizures
Garvey et al. 1998	Retrospective study	US	July 1993 to June 1994	107	Pediatrics	CT in the emergency department	Children with a “first time seizure”
Machingaidze et al. 2020	Retrospective observational study	South Africa	January 1, 2013 to December 31, 2013	311	Pediatrics	Head CT scan within 24 h	1. Seizures (n = 169; 54.3%) < br > 2. Reduced level of consciousness (n = 140; 45.0%) < br > 3. Headache (n = 74; 23.8%) < br > 4. Suspected ventriculoperitoneal shunt (VPS) malfunction (n = 61; 19.7%)
Veerapandiyan et al. 2018	Retrospective, cross-sectional study	US	2010 to 2014	155	Pediatrics	CT head	New-Onset Afebrile Seizure
Tan et al. 2021	Retrospective study	Singapore	January 1, 2015 to December 31, 2018	479	Pediatrics	CT head	1. Seizure (149, 31.1%) < br > 2. Head injury (147, 30.7%) < br > 3. Altered mental status (44, 9.2%)
Maytal et al. 2000	Retrospective study	US	January 1 to December 31, 1995	66	Pediatrics	CT brain	Diagnostic utility of emergency brain CT in children presenting with new onset of seizures
Al-Rumayyan et al. 2012	Cross-sectional study	Saudi Arabia	January 2005 to December 2010	124	Pediatrics	CT brain	Pediatric patients presenting with a first seizure
Bautovich et al. 2012	Retrospective study	Australia	November 2005 to September 2009	89	Pediatrics	CT brain	Diagnosis of first episode of seizure
Brugman et al. 2019	Retrospective cross-sectional study	South Africa	January 1, 2013 to August 31, 2018	468	Pediatrics	CT brain	First episode seizure
Cohen et al. 2018	Retrospective chart review	US	January 2011 to December 2015	408	Pediatrics	CT brain	Seizure in children with intraventricular shunt for hydrocephalus
Garg et al. 1997	Observational study	India	Not specified	121	Pediatrics	CT brain	First seizures in children aged 6 to 15 years
Lyons et al. 2016	Retrospective cohort study	US	October 1995 to September 2012	177	Pediatrics	CT brain	Children with new-onset seizures presenting with status epilepticus

*CTH* Computed Tomography of the Head, *CT* Computed Tomography, *VPS* Ventriculoperitoneal Shunt, *US* United States, *AMS* Altered Mental Status

**Table 2:** Summary of included studies on CTH utilization and positive findings

Study ID	Groups (Interventions/Exposures)	Age	Sex (Male/Female)	Event Rate of CTH Utilization in Acute Atraumatic AMS	Event Rate of Positive CTH	Sample Size
**Allen et al. 2007**	Computed tomography of the head (CTH)	Mean: 8.8 years (SD 4.5)	Ratio 1.2:1	21	4	124
**Cavallaro et al. 2021**	CT at pediatric emergency department	Not mentioned	Not mentioned	1.21 million	Not mentioned	5.41 million
	CT at general emergency department	Not mentioned	Not mentioned	192,357	Not mentioned	1.45 million
**Garvey et al. 1998**	CT in the emergency department	Not mentioned	Not mentioned	99	19	107
**Machingaidze et al. 2020**	Head CT scan within 24 h of presentation with a non-trauma event	Median: 39.2 months (IQR: 12.6–84)	188/123	311	92	311
**Veerapandiyan et al. 2018**	CT head	Not mentioned	88/67	72	9	155
**Tan et al. 2021**	CT brain	Median: 7 years (IQR: 3–12)	290/67	332	332	479
**Maytal et al. 2000**	CT brain	Mean: 4.9 years	34/32	66	14	66
**Al-Rumayyan et al. 2012**	CT brain	Mean: 3.5 years	74/50	124	53	124
**Bautovich et al. 2012**	CT brain	Median: 2.5 years	Not mentioned	71	16	89
**Brugman et al. 2019**	CT brain	Median: 27 months (IQR: 12–68.3)	228/240	468	184	468
**Cohen et al. 2018**	CT brain	Median: 6 years (IQR: 3–11)	Not mentioned	318	32	408
**Garg et al. 1997**	CT brain	Mean: 9.24 years (SD 3.89)	102/19	94	121	121
**Lyons et al. 2016**	CT brain	Median: 9.1 years (IQR: 1.6–14)	9/6	177	64	15 (urgent intracranial pathology)
		Median: 2.1 years (IQR: 1.2–4.7)	97/65			162 (not urgent intracranial pathology)

*AMS* Acute Atraumatic Altered Mental Status, *CTH* Computed Tomography of the Head, *CT* Computed Tomography, *ED* Emergency Department

**Fig. 2:**
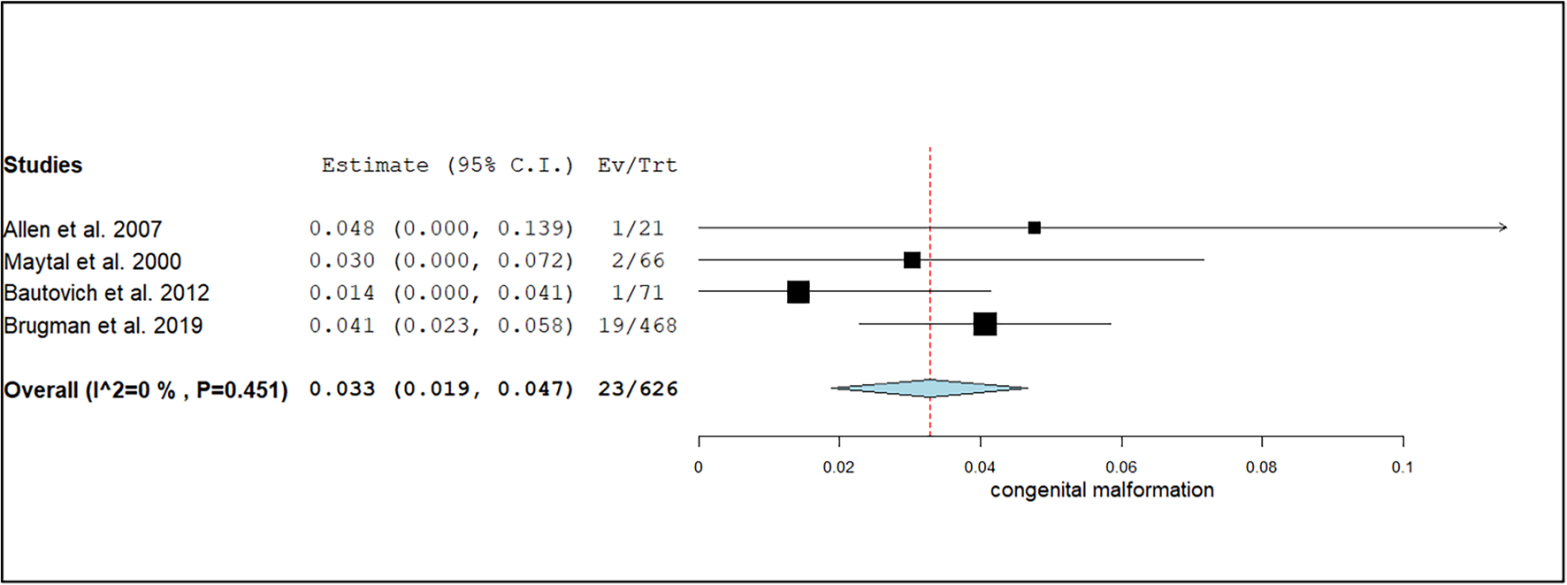
Forest plot of the event rate for the diagnosis of congenital malformations using CT head

**Fig. 3:**
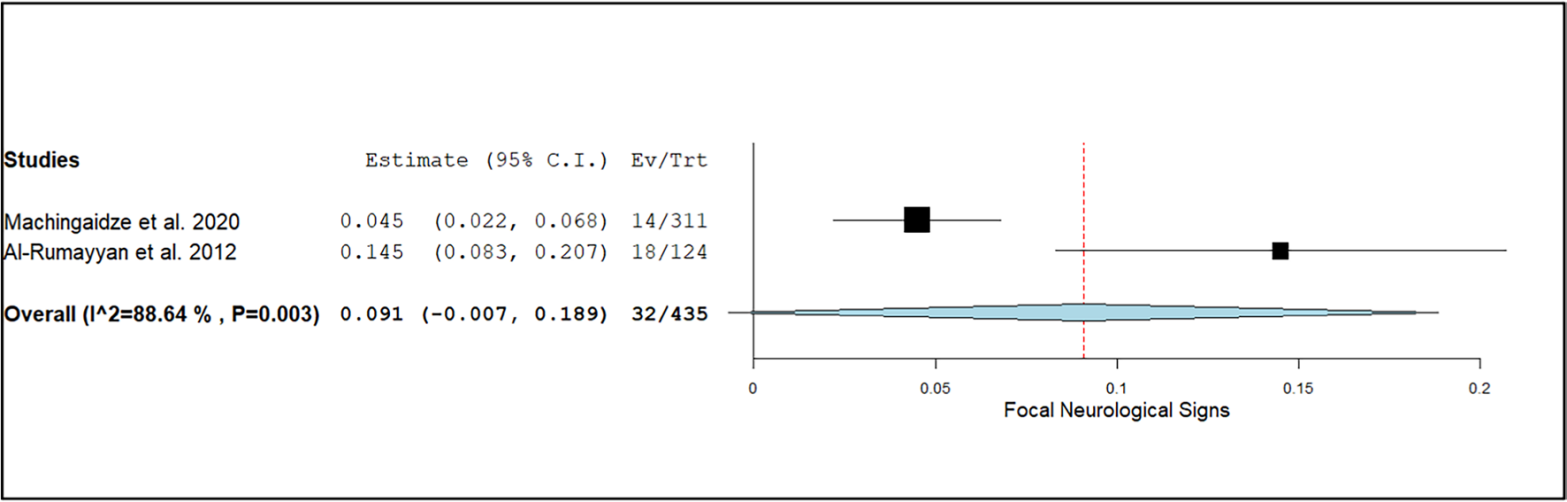
Forest plot of the event rate for the diagnosis of focal neurological deficits using CT head

**Fig. 4:**
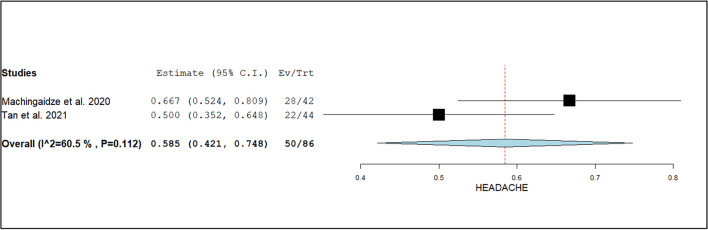
Forest plot of the rate of usage of CT scan of the head in patients with headache

The diagnostic yield of CT scans was stratified by various factors. The event rate for the diagnosis of congenital malformations was 3.3% (95% CI: 1.9%–4.7%; I^2^ = 28.2%, *p* = 0.12), as shown in Fig. 2. For focal neurological deficits, the diagnostic yield was higher at 9.1% (95% CI: −0.7%–18.9%; I^2^ = 82%, *p* < 0.01), as illustrated in Fig. 3. Patients presenting with headaches had a diagnostic yield of 5.8% (95% CI: 42.1%–74.8%; I^2^ = 91%, *p* < 0.01), as seen in Fig. 4.

**Fig. 5:**
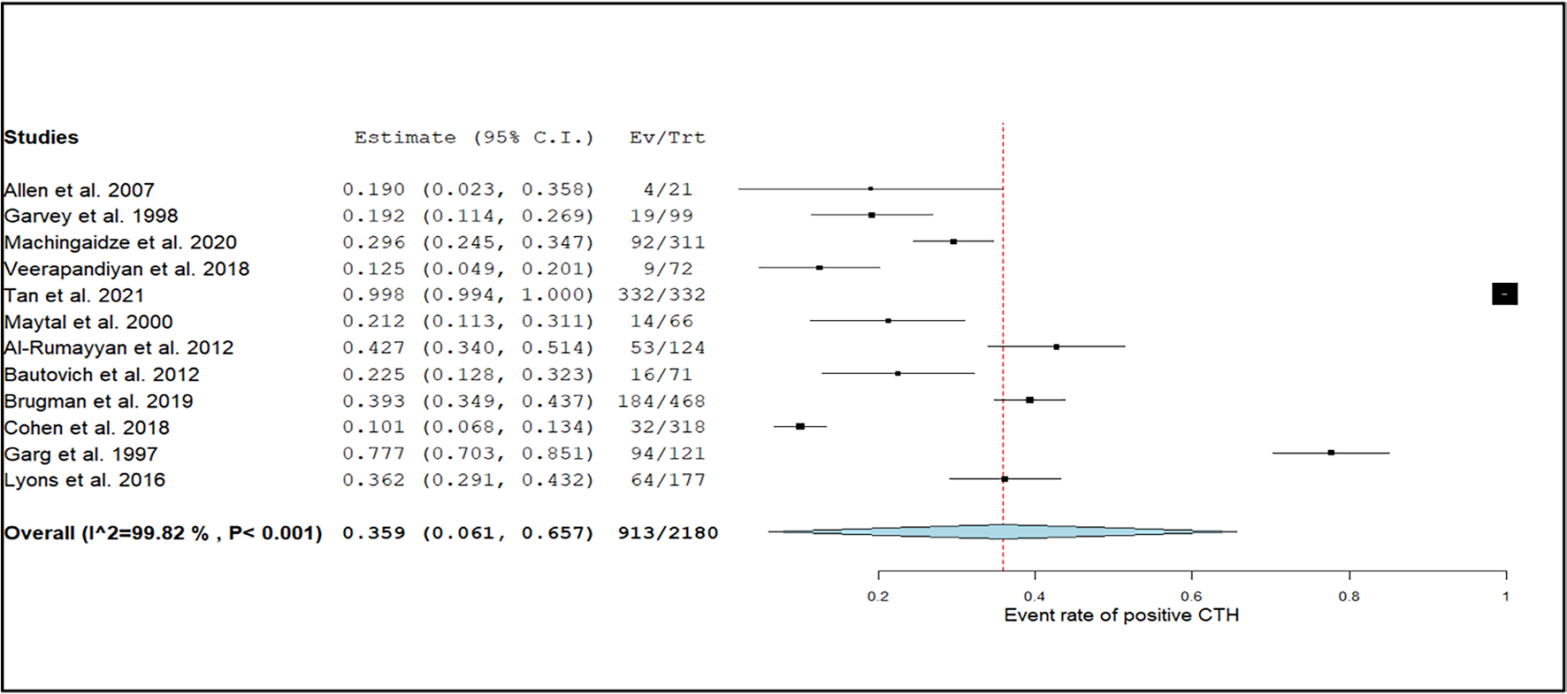
Forest plot of the event rate of positive CTH

The overall positive diagnostic yield of head CT scans was 35.9% (95% CI: 6.1%–65.7%), with significant heterogeneity noted (I^2^ = 99%, *p* < 0.001), as shown in Fig. 5. Among the included studies, the diagnostic yield was higher in inpatient settings (45%; 95% CI: 30.2%–59.8%; I^2^ = 85%, *p* < 0.01) compared to emergency departments (28%; 95% CI: 15.4%–40.6%; I^2^ = 72%, *p* = 0.02), likely reflecting the selection of more severe or complex cases in inpatient care.

**Fig. 6:**
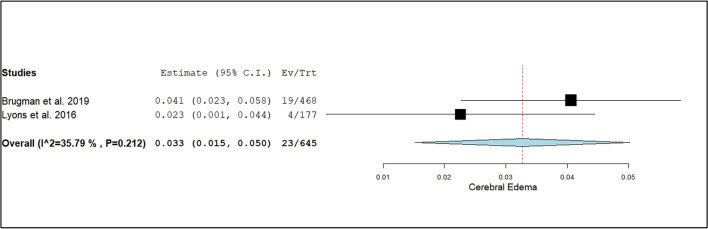
Forest plot of the event rate for the diagnosis of cerebral edema using CT head

Other notable findings included the diagnosis of cerebral edema, with a rate of 3.3% (95% CI: 1.5%–5.0%; I^2^ = 35.8%, *p* = 0.21), as shown in Fig. 6. Hydrocephalus was observed at a rate of 4.3% (95% CI: 0.8%–7.7%; I^2^ = 40.1%, *p* = 0.19). Additional outcomes, including rates for intracranial hemorrhage, neoplasms, and strokes, are summarized in Table 3.

**Table 3:** Rate and incidence of outcomes and the diagnostic yield of head CT scans in pediatric AMS

Outcome	Rate/Incidence	Confidence Interval
Overall Positive Event Rate (Head CT scans)	35.9%	(95% CI: 6.1%–65.7%)
Cerebral Edema	3.3%	(95% CI: 1.5%–5.0%)
Congenital Malformations	3.3%	(95% CI: 1.9%–4.7%)
Focal Neurological Deficits	9.1%	(95% CI: −0.7%–18.9%)
Seizures	27.3%	(95% CI: 8.9%–45.7%)
Headaches	5.8%	(95% CI: 42.1%–74.8%)
Hydrocephalus	4.3%	(95% CI: 0.8%–7.7%)
Intracranial Hemorrhage	1.8%	(95% CI: 0.7%–2.9%)
Meningoencephalitis	3.6%	(95% CI: −1.6%–8.8%)
Neoplasms	2.9%	(95% CI: 1.4%–4.3%)
Stroke	5.6%	(95% CI: −3.4%–14.5%)

Unspecified outcomes were reported in 10 studies, with an unspecified outcome rate of 15.2% (95% CI: 8.4%–21.9%; I^2^ = 99%, *p* < 0.001), reflecting considerable variability in outcome definitions across studies (Supplementary Figure S9).

The risk of bias was assessed using the ROBINS-I tool, which indicated that most included studies exhibited low risk of bias across various domains. However, some studies showed moderate risk in areas such as participant selection and handling of missing data. One study exhibited serious risk of bias due to incomplete outcome reporting. The risk of bias assessment is summarized in Supplementary Figure S10.

## Discussion

This meta-analysis provides valuable insights into the diagnostic utility and clinical outcomes of head CT scans in pediatric patients with acute atraumatic altered mental status (AMS). The findings highlight the prevalent use of head CT imaging, despite its relatively low diagnostic yield, which raises concerns about overutilization and associated risks [16–21]. The pooled positive event rate of 35.9% indicates that head CT scans reveal significant findings in only about one-third of pediatric AMS cases, suggesting that many children undergo imaging without meaningful diagnostic outcomes. This exposes patients to unnecessary radiation, which increases the lifetime risk of malignancies, particularly brain tumors, due to the heightened sensitivity of developing tissues [3, 4]. Additionally, unnecessary imaging contributes to rising healthcare costs without proportional clinical benefits [10].

Our analysis revealed significant variability in the diagnostic yield of CT scans based on clinical settings, patient demographics, and presenting symptoms. Inpatient settings demonstrated a higher diagnostic yield (45%) compared to emergency departments (28%), likely reflecting the selection of more severe or complex cases in inpatient care. When stratified by age, infants under one year of age exhibited the highest positive yield (42%), followed by children aged 1–12 years (35%) and adolescents aged 13–18 years (30%). Regarding presenting symptoms, patients with focal neurological deficits had the highest diagnostic yield (60%), underscoring the importance of thorough neurological assessments. Seizures were also strong predictors of abnormal findings, with a 40% positive rate, whereas headaches were associated with a lower diagnostic yield of 20% [19, 21–24].

These findings emphasize the need for validated risk stratification tools to better identify patients most likely to benefit from imaging, prioritizing CT scans for high-risk groups while reducing unnecessary radiation exposure in low-risk patients [25, 26].

### Comparison with existing literature

Our findings are consistent with previous research. For example, Jahanshahi et al. reported that 68.5% of CT scans in a pediatric cohort were normal, with hydrocephalus identified in 13.9% of cases and benign infantile hydrocephalus in 7.4% [21]. Similarly, Bautovich et al. observed that CT findings influenced acute management in only 7% of pediatric seizure cases, suggesting limited utility in routine imaging [19]. A separate meta-analysis observed a CT utilization rate of 94% in AMS patients but a positive event rate of only 11%, underscoring the disconnect between frequent imaging and its limited diagnostic yield [13]. These findings align with our results, further highlighting the need for more targeted imaging approaches.

### Clinical implications

The variability in diagnostic yield and overutilization of CT imaging underscore the need for more strategic approaches in clinical practice. Developing validated risk stratification tools to guide imaging decisions is crucial, particularly for high-risk patients such as those with focal neurological deficits or seizures. Comprehensive history-taking and physical examinations remain fundamental for identifying patients most likely to benefit from imaging. Adherence to evidence-based guidelines, which recommend imaging only in specific scenarios (e.g., prolonged postictal states or abnormal neurological examinations), is critical for optimizing CT utilization and reducing exposure to ionizing radiation [10, 26, 27].

### Strengths and future directions

This study is the first comprehensive meta-analysis evaluating head CT scans in pediatric AMS across diverse healthcare settings. Its large sample size enhances the generalizability of the findings. However, substantial heterogeneity among the included studies limits the precision of subgroup analyses, particularly those related to specific neurological findings. Future research should prioritize the development and validation of risk stratification models for pediatric AMS to enable more targeted imaging decisions. Prospective studies are also needed to establish standardized imaging protocols and evaluate alternative imaging modalities, such as MRI or ultrasound, which may reduce ionizing radiation risks [4, 28].

### Limitations

This study has several limitations. The heterogeneity among included studies, such as differences in study design and patient populations, affects the generalizability of findings. Publication bias may also be present, as studies with negative or non-significant results are likely underrepresented. Furthermore, data gaps restricted the exploration of some associations, and the retrospective nature of most included studies introduces potential biases in data collection and interpretation [13, 24].

### Conclusion

Head CT scans remain a valuable diagnostic tool in pediatric AMS; however, their moderate diagnostic yield and associated risks necessitate cautious and selective application. The findings highlight the importance of implementing decision-support systems and adhering to evidence-based clinical guidelines to optimize imaging practices. Future multicenter collaborations are crucial to refining imaging strategies and improving outcomes for this vulnerable population.

## Supplementary Information

Below is the link to the electronic supplementary material.

Supplementary file1 (DOCX 14 KB)

Supplementary file2 (DOCX 521 KB)

## Data Availability

No datasets were generated or analysed during the current study.

## Abbreviations

CT: Computed Tomography
AMS: Altered Mental Status
ROBINS-I: Risk of Bias in Non-randomized Studies of Interventions
CTH: Computed Tomography of the Head

## References

[1] Kvam KA, Douglas VC, Whetstone WD et al (2019) Yield of Emergent CT in Patients With Epilepsy Presenting With a Seizure. Neurohospitalist 9:71. 10.1177/1941874418808676 30915184 10.1177/1941874418808676 PMC6429671

[2] Maytal J, Krauss JM, Novak G et al (2000) The role of brain computed tomography in evaluating children with new onset of seizures in the emergency department. Epilepsia 41:950–954. 10.1111/J.1528-1157.2000.TB00277.X 10961619 10.1111/j.1528-1157.2000.tb00277.x

[3] Power SP, Moloney F, Twomey M et al (2016) Computed tomography and patient risk: Facts, perceptions and uncertainties. World J Radiol 8:902. 10.4329/WJR.V8.I12.902 28070242 10.4329/wjr.v8.i12.902 PMC5183924

[4] Ferrero A, Takahashi N, Vrtiska TJ et al (2019) Understanding, justifying, and optimizing radiation exposure for CT imaging in nephrourology. Nat Rev Urol 16:231. 10.1038/S41585-019-0148-8 30728476 10.1038/s41585-019-0148-8 PMC6447446

[5] Ulsh BA (2015) Are Risks From Medical Imaging Still too Small to Be Observed or Nonexistent? Dose-Response 13. 10.2203/DOSE-RESPONSE.14-030.ULSH 10.2203/dose-response.14-030.Ulsh PMC4674180 26673121

[6] Thierry-Chef I, Simon SL, Land CE, Miller DL (2008) Radiation dose to the brain and subsequent risk of developing brain tumors in pediatric patients undergoing interventional neuroradiology procedures. Radiat Res 170:553. 10.1667/RR1393.1 18959462 10.1667/RR1393.1 PMC4018570

[7] Kim SK, Jung JH, Lee JH et al (2019) The difference of Use of CT in the general versus pediatric emergency departments for adolescent patients in the same tertiary hospital. Clin Exp Emerg Med 6:19. 10.15441/CEEM.17.274 30786703 10.15441/ceem.17.274 PMC6453689

[8] Prevalence and prediction of abnormal CT scan in pediatric patients presenting with a first seizure - PubMed. https://pubmed.ncbi.nlm.nih.gov/23022900/. Accessed 11 Feb 2024 23022900

[9] Bautovich T, Numa A (2012) Role of head computed tomography in the evaluation of children admitted to the paediatric intensive care unit with new-onset seizure. EMA - Emerg Med Australasia 24:313–320. 10.1111/j.1742-6723.2012.01561.x 10.1111/j.1742-6723.2012.01561.x 22672172

[10] American Academy of Neurology: Neurology Resources | AAN. https://www.aan.com/PressRoom/home/PressRelease/553. Accessed 11 Feb 2024

[11] Allen L, Jones CT (2007) Emergency department use of computed tomography in children with epilepsy and breakthrough seizure activity. J Child Neurol 22:1099–1101. 10.1177/0883073807306249 17890407 10.1177/0883073807306249

[12] Machingaidze PR, Buys H, Kilborn T, Muloiwa R (2020) Clinical use and indications for head computed tomography in children presenting with acute medical illness in a low- and middle-income setting. PLoS One 15. 10.1371/JOURNAL.PONE.0239731 10.1371/journal.pone.0239731 PMC7521723 32986760

[13] Page MJ, McKenzie JE, Bossuyt PM et al (2021) The PRISMA 2020 statement: an updated guideline for reporting systematic reviews. BMJ 372. 10.1136/BMJ.N71 10.1136/bmj.n71 PMC8005924 33782057

[14] Cumpston MS, McKenzie JE, Welch VA, Brennan SE (2022) Strengthening systematic reviews in public health: guidance in the Cochrane Handbook for Systematic Reviews of Interventions, 2nd edition. J Public Health (Oxf) 44:E588–E592. 10.1093/PUBMED/FDAC036 10.1093/pubmed/fdac036 PMC9715291 35352103

[15] Acharya R, Kafle S, Shrestha DB et al (2022) Use of computed tomography of the head in patients with acute atraumatic altered mental status: a systematic review and meta-analysis. JAMA Netw Open 5:E2242805. 10.1001/JAMANETWORKOPEN.2022.42805 36399344 10.1001/jamanetworkopen.2022.42805 PMC9675006

[16] MollaMohammadi M, Tonekaboni SH, Khatami A et al (2013) Neuroimaging findings in first unprovoked seizures: a multicentric study in Tehran. Iran J Child Neurol 7:24 24665314 PMC3943050

[17] Lyons TW, Johnson KB, Michelson KA et al (2016) Yield of emergent neuroimaging in children with new-onset seizure and status epilepticus. Seizure 35:4–10. 10.1016/J.SEIZURE.2015.12.009 26773658 10.1016/j.seizure.2015.12.009 PMC5369645

[18] Cohen A, Agarwal R, Farooqi A, Kannikeswaran N (2018) Is shunt evaluation useful in children with intraventricular shunts with seizures? Pediatr Neurol 88:59–64. 10.1016/J.PEDIATRNEUROL.2018.08.005 30327239 10.1016/j.pediatrneurol.2018.08.005

[19] Bautovich T, Numa A (2012) Role of head computed tomography in the evaluation of children admitted to the paediatric intensive care unit with new-onset seizure. Emerg Med Australas 24:313–320. 10.1111/J.1742-6723.2012.01561.X 22672172 10.1111/j.1742-6723.2012.01561.x

[20] Brugman J, Solomons RS, Lombard C et al (2020) Risk-stratification of children presenting to ambulatory paediatrics with first-onset seizures: should we order an urgent CT brain? J Trop Pediatr 66:299–314. 10.1093/TROPEJ/FMZ071 31625577 10.1093/tropej/fmz071

[21] Jahanshahi A, Sadeghvand S, Khalafi M et al (2023) Prevalence of positive findings of brain computed tomography scans in pediatric population. Iran J Child Neurol 17:111. 10.22037/IJCN.V17I1.36227 10.22037/ijcn.v17i1.36227 PMC10114275 37091465

[22] OpenMeta[Analyst]. http://www.cebm.brown.edu/openmeta/download.html. Accessed 11 Feb 2024

[23] Cavallaro SC, Monuteaux MC, Chaudhari PP, Michelson KA (2021) Use of neuroimaging for children with seizure in general and pediatric emergency departments. J Emerg Med 60:478. 10.1016/J.JEMERMED.2020.10.044 33419652 10.1016/j.jemermed.2020.10.044 PMC8084929

[24] (UK) NGA (2022) Computed tomography scan performance in people with epilepsy. Computed tomography scan performance in people with epilepsy: Epilepsies in children, young people and adults: Evidence review B 35700285

[25] Sartori S, Nosadini M, Tessarin G et al (2019) First-ever convulsive seizures in children presenting to the emergency department: risk factors for seizure recurrence and diagnosis of epilepsy. Dev Med Child Neurol 61:82–90. 10.1111/DMCN.14015/ABSTRACT 30191957 10.1111/dmcn.14015

[26] Sterne JAC, Hernán MA, Reeves BC, Savović J, Berkman ND, Viswanathan M, Henry D et al (2016) The Risk Of Bias In Non-Randomized Studies of Interventions ( ROBINS-I ). BMJ 355:i4919 27733354 10.1136/bmj.i4919 PMC5062054

[27] Jiménez-Villegas MJ, Lozano-García L, Carrizosa-Moog J (2021) Update on first unprovoked seizure in children and adults: A narrative review. Seizure 90:28–33. 10.1016/J.SEIZURE.2021.03.027 33840584 10.1016/j.seizure.2021.03.027

[28] Gaillard WD, Chiron C, Helen Cross J et al (2009) Guidelines for imaging infants and children with recent-onset epilepsy. Epilepsia 50:2147–2153. 10.1111/J.1528-1167.2009.02075.X 19389145 10.1111/j.1528-1167.2009.02075.x

