# Supplementary material for: Diagnostic value of CT scans in pediatric patients with acute non-traumatic altered mental status: a systematic review and meta-analysis
Source: Eur J Pediatr. 2025 Jan 15;184(2):136. doi: 10.1007/s00431-024-05943-3 (PMC11735565; doi:10.1007/s00431-024-05943-3)
Supplement: Supplementary file 1 — Supplementary file1 (DOCX 14 KB) [file 431_2024_5943_MOESM1_ESM.docx]

| Database | Strategy | Results |
| --- | --- | --- |
| PubMed | ("CT scan" OR "Computed tomography") AND ("Children" OR "Pediatric" OR "Pediatrics" OR "Adolescents" OR "Infants") AND ("Altered mental status" OR "Altered consciousness" OR "Confusion" OR "Disorientation" OR "Encephalopathy") | 852 |
| SCOPUS | TITLE-ABS-KEY("CT scan" OR "Computed tomography") AND TITLE-ABS-KEY("Children" OR "Pediatric" OR "Pediatrics" OR "Adolescents" OR "Infants") AND TITLE-ABS-KEY("Altered mental status" OR "Altered consciousness" OR "Confusion" OR "Disorientation" OR "Encephalopathy") | 1598 |
| Cochrane Library | ("CT scan" OR "Computed tomography") AND ("Children" OR "Pediatric" OR "Pediatrics" OR "Adolescents" OR "Infants") AND ("Altered mental status" OR "Altered consciousness" OR "Confusion" OR "Disorientation" OR "Encephalopathy") | 25 |
| Embase | ('CT scan' OR 'Computed tomography') AND ('Child' OR 'Pediatric' OR 'Pediatrics' OR 'Adolescent' OR 'Infant') AND ('Altered mental status' OR 'Altered consciousness' OR 'Confusion' OR 'Disorientation' OR 'Encephalopathy') | 1791 |
| Web of Science | TS=("CT scan" OR "Computed tomography") AND TS=("Children" OR "Pediatric" OR "Pediatrics" OR "Adolescents" OR "Infants") AND TS=("Altered mental status" OR "Altered consciousness" OR "Confusion" OR "Disorientation" OR "Encephalopathy") | 473 |

Date: 23-5-2024

Research Strategy (Table S).
