# Supplementary material for: Diagnostic value of CT scans in pediatric patients with acute non-traumatic altered mental status: a systematic review and meta-analysis
Source: Eur J Pediatr. 2025 Jan 15;184(2):136. doi: 10.1007/s00431-024-05943-3 (PMC11735565; doi:10.1007/s00431-024-05943-3)
Supplement: Supplementary file 2 — Supplementary file2 (DOCX 521 KB) [file 431_2024_5943_MOESM2_ESM.docx]

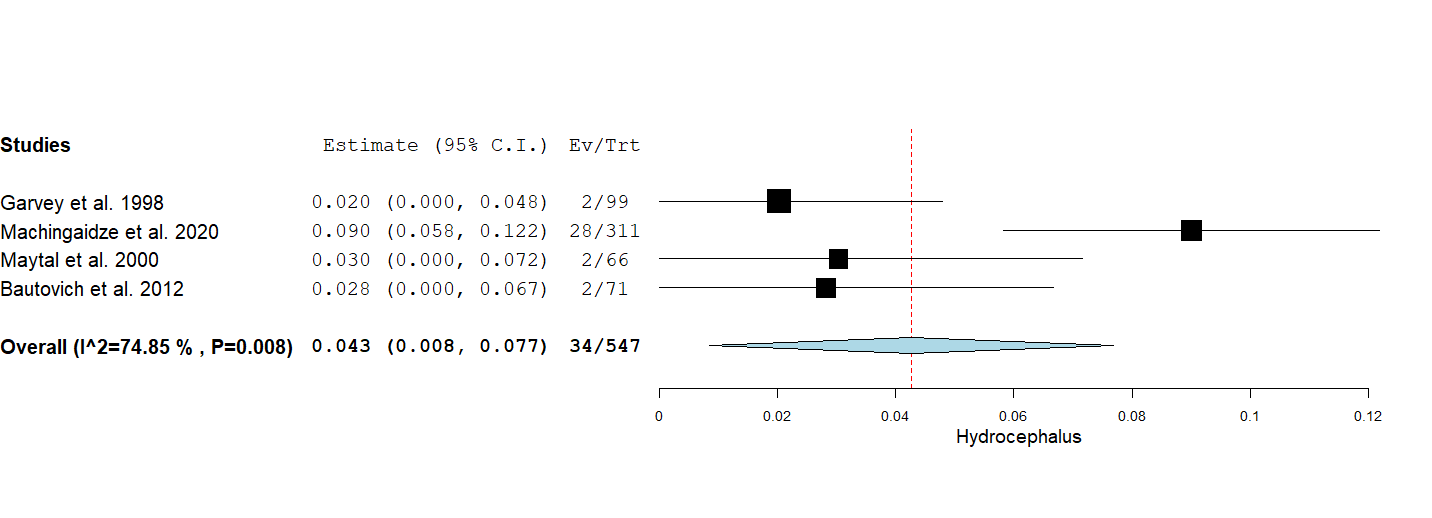


**Figure S1:** Forest plot of the rate of usage of CT scan of the head in patients with hydrocephalus.


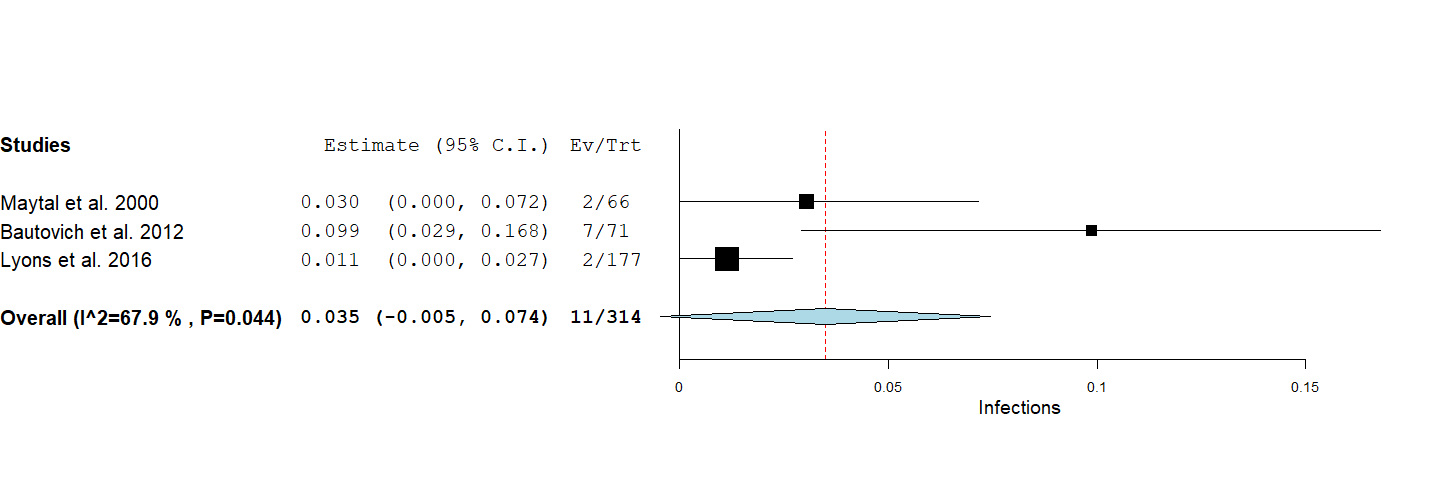


**Figure S2:** Forest plot of the rate of usage of CT scan of the head in patients with infection.


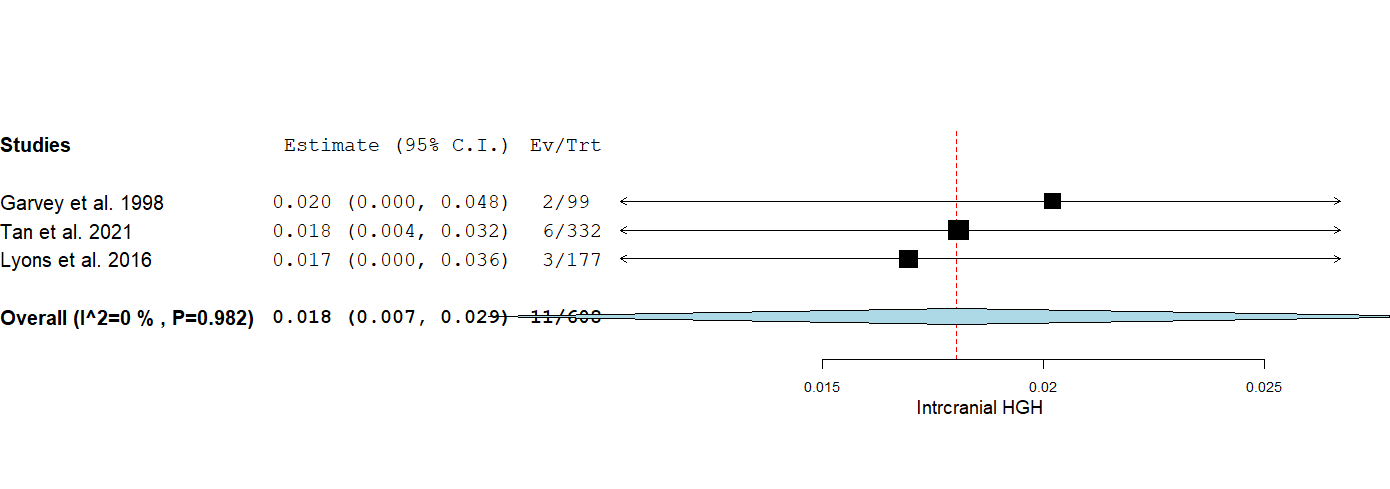


**Figure S3:** Forest plot of the rate of usage of CT scan of the head in patients with intracranial hemorrhage.

**
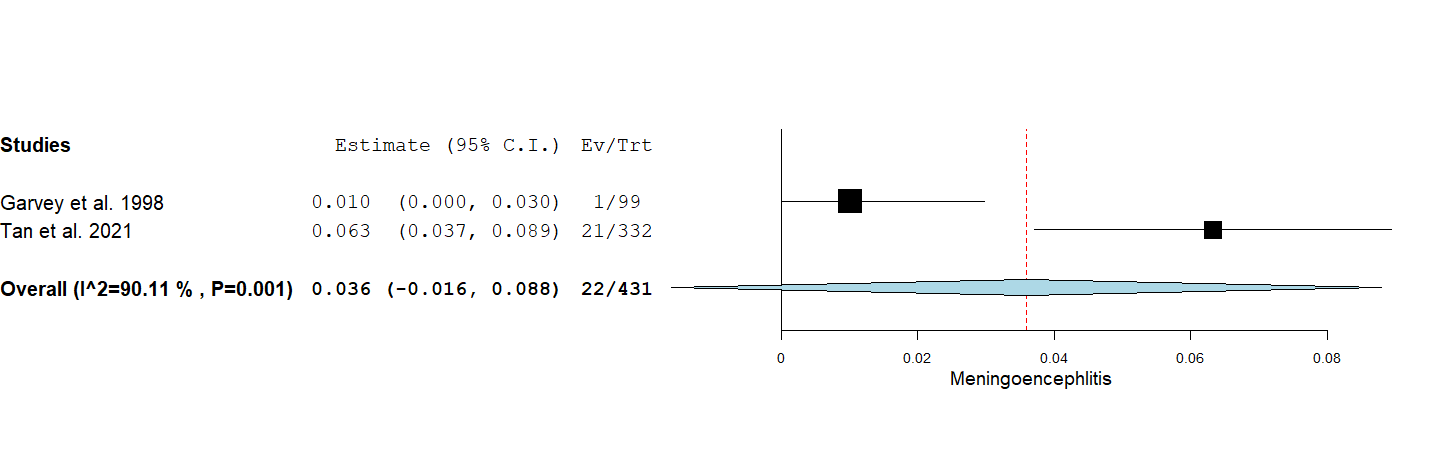
**

**Figure S4:** Forest plot of the rate of usage of CT scan of the head in patients with meningoencephalitis presentation.


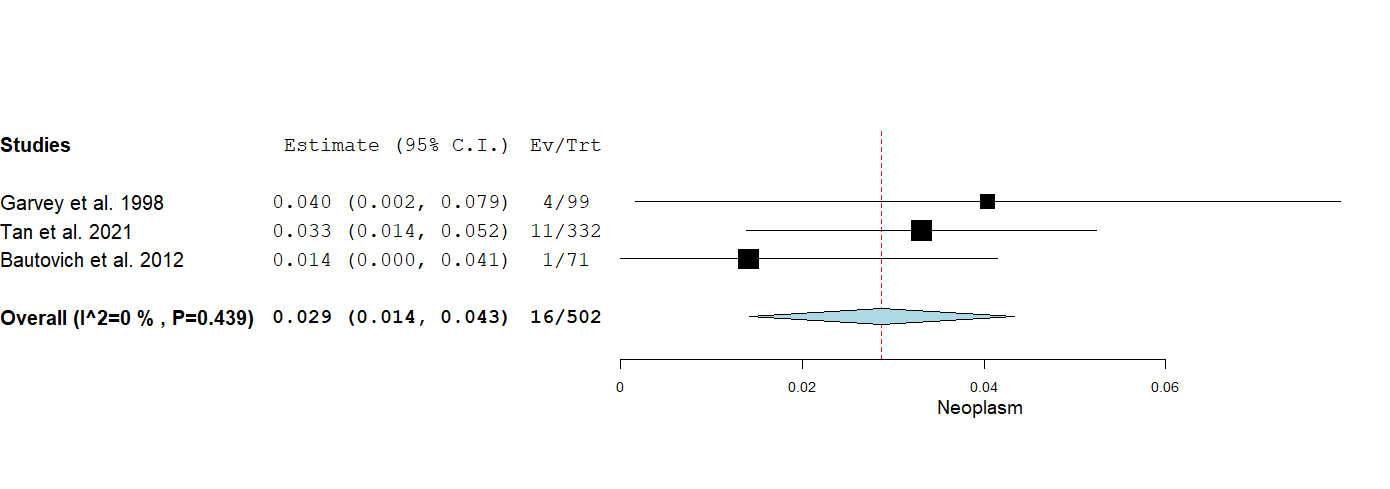


**Figure S5:** Forest plot of the rate of usage of CT scan of the head to aid in the diagnosis of neoplasms.


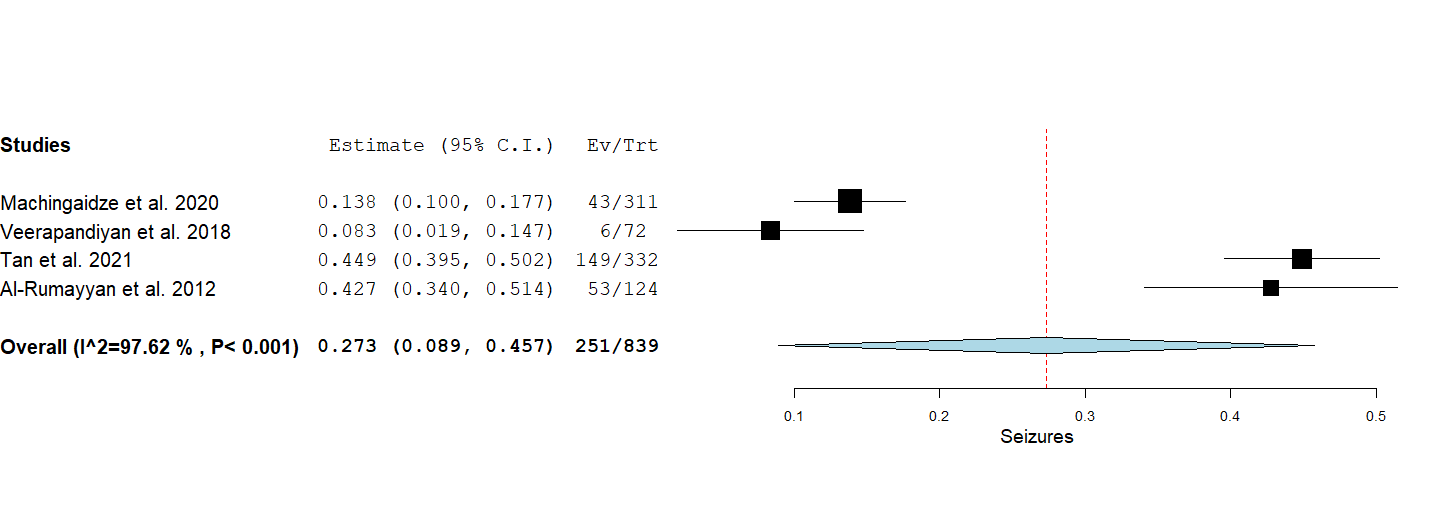


**Figure S6:** Forest plot of the rate of usage of CT scan of the head in patients with seizures.


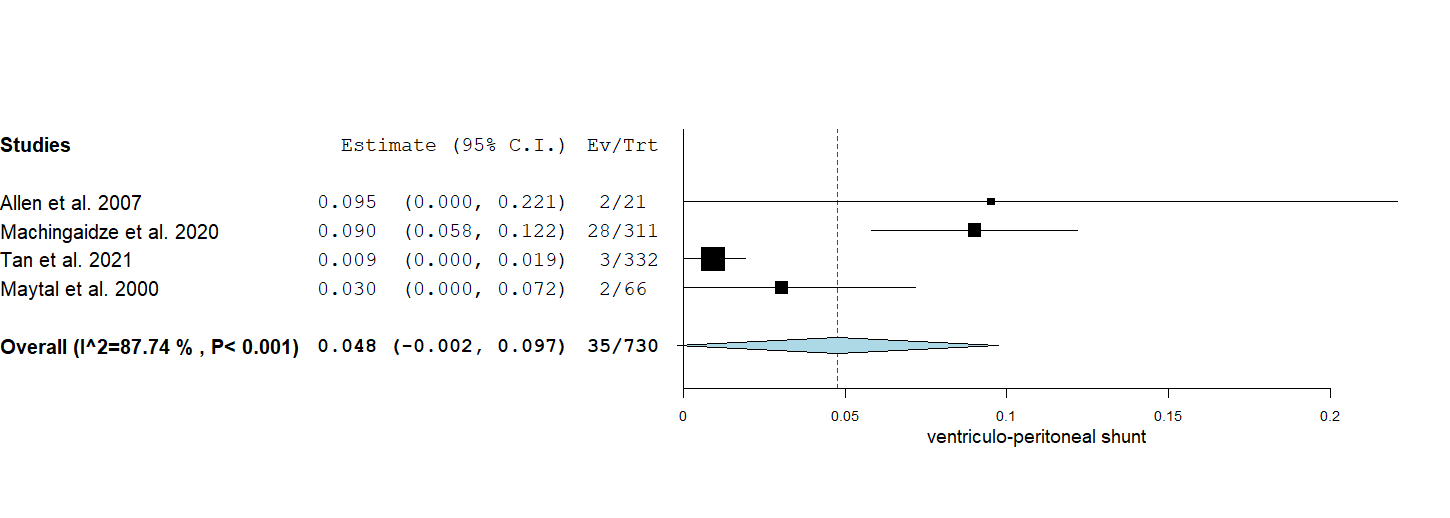


**Figure S7:** Forest plot of the rate of usage of CT scan of the head in patients with VP shunts.


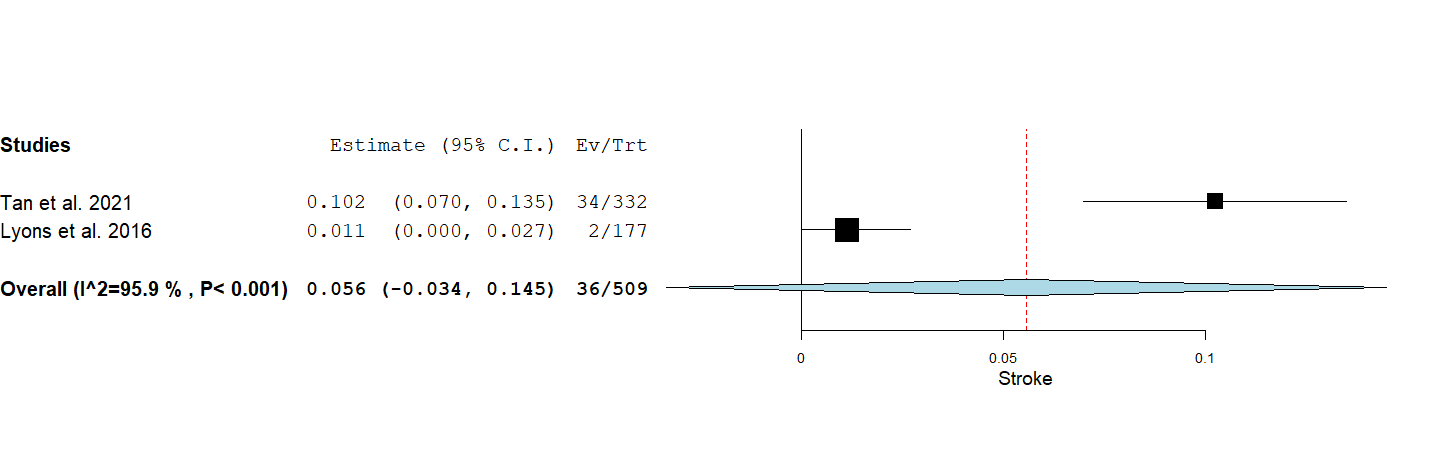


**Figure S8:** Forest plot of the rate of usage of CT scan of the head to aid in the diagnosis of stroke.


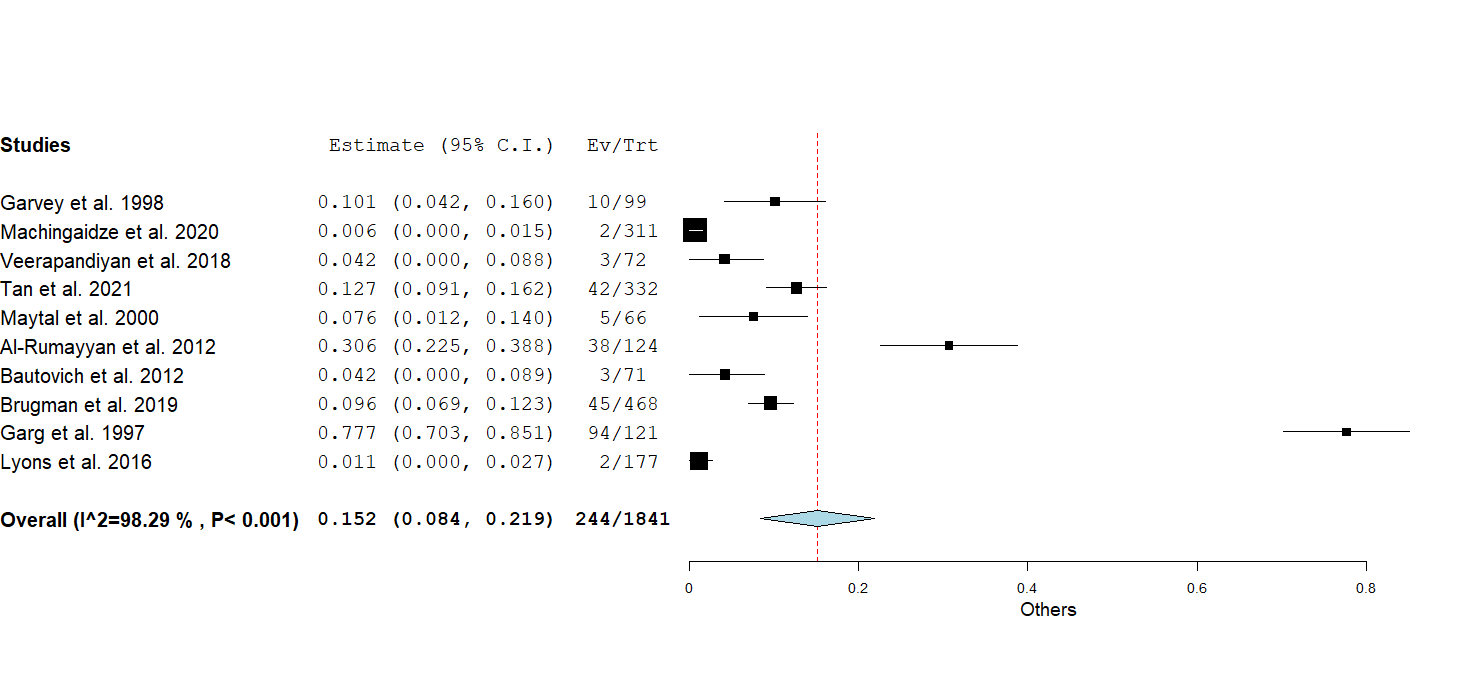


**Figure S9:** Forest plot of the rate of usage of CT scan of the head for other non-specified reasons which are not specifically mentioned in the literature.


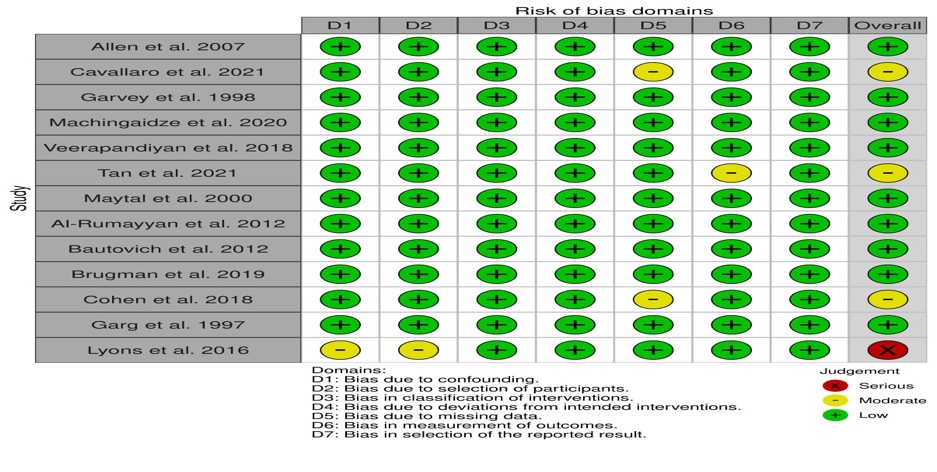


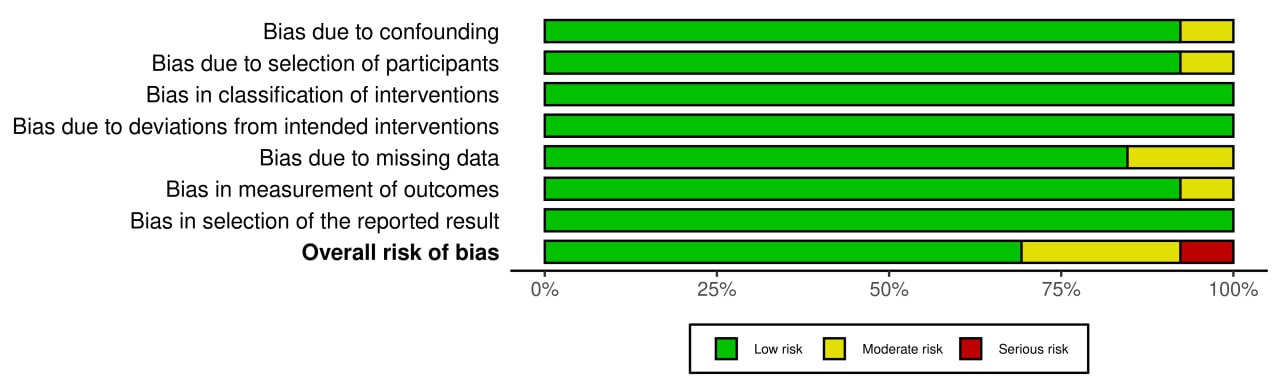


**Fig.S10**: Risk of bias assessment using the ROBINS-I tool presented as a proportion of relevant studies (n = 13)

**Figures’ Legends**

**Fig.S1:** Forest plot of the rate of usage of CT scan of the head in patients with hydrocephalus.

**Fig.S2:** Forest plot of the rate of usage of CT scan of the head in patients with infection.

**Fig.S3:** Forest plot of the rate of usage of CT scan of the head in patients with intracranial hemorrhage.

**Fig.S4:** Forest plot of the rate of usage of CT scan of the head in patients with meningoencephalitis presentation.

**Fig. S5:** Forest plot of the rate of usage of CT scan of the head to aid in the diagnosis of neoplasms.

**Fig.S6:** Forest plot of the rate of usage of CT scan of the head in patients with seizures.

**Fig.S7:** Forest plot of the rate of usage of CT scan of the head in patients with VP shunts.

**Fig.S8:** Forest plot of the rate of usage of CT scan of the head to aid in the diagnosis of stroke.

**Fig.S9:** Forest plot of the rate of usage of CT scan of the head for other non-specified reasons which are not specifically mentioned in the literature.

**Fig. S10**: Risk of bias assessment using the ROBINS-I tool presented as a proportion of relevant studies (n = 13)
